# Intake of bean fiber, beans, and grains and reduced risk of hormone receptor‐negative breast cancer: the San Francisco Bay Area Breast Cancer Study

**DOI:** 10.1002/cam4.1423

**Published:** 2018-03-23

**Authors:** Meera Sangaramoorthy, Jocelyn Koo, Esther M. John

**Affiliations:** ^1^ Cancer Prevention Institute of California Fremont California 94538; ^2^ Department of Health Research and Policy (Epidemiology) and Stanford Cancer Institute Stanford University School of Medicine Stanford Stanford California 94305

**Keywords:** African Americans, beans, breast cancer, diet, fiber, grains, Hispanics

## Abstract

High dietary fiber intake has been associated with reduced breast cancer risk, but few studies considered tumor subtypes defined by estrogen receptor (ER) and progesterone receptor (PR) status or included racial/ethnic minority populations who vary in their fiber intake. We analyzed food frequency data from a population‐based case–control study, including 2135 breast cancer cases (1070 Hispanics, 493 African Americans, and 572 non‐Hispanic Whites (NHWs)) and 2571 controls (1391 Hispanics, 557 African Americans, and 623 NHWs). Odds ratios (OR) and 95% confidence intervals (CI) for breast cancer associated with fiber intake were calculated using unconditional logistic regression. Breast cancer risk associated with high intake (high vs. low quartile) of bean fiber (p‐trend = 0.01), total beans (p‐trend = 0.03), or total grains (p‐trend = 0.05) was reduced by 20%. Inverse associations were strongest for ER‐PR‐ breast cancer, with risk reductions associated with high intake ranging from 28 to 36%. For bean fiber, risk was reduced among foreign‐born Hispanics only, who had the highest fiber intake, whereas for grain intake, inverse associations were found among NHWs only. There was no evidence of association with fiber intake from vegetables and fruits or total intake of vegetables and fruits. A high dietary intake of bean fiber and fiber‐rich foods such as beans and grains may lower the risk of ER‐PR‐ breast cancer, an aggressive breast cancer subtype for which few risk factors have been identified.

## Introduction

The role of dietary fiber in breast cancer etiology remains uncertain 1, 2. Meta‐analyses of cohort 2, 3, 4 and case–control 5 data have reported inverse associations, with modest risk reductions of 5%‐7% per 10 g/day increment of fiber intake. In some studies, associations with fiber intake varied by source of fiber (e.g., vegetables, fruits, and grains) 6, 7, 8, 9, 10, 11, 12, 13, 14 or type of fiber (e.g., soluble vs. insoluble) 15, 16. Recent studies also suggest that associations with fiber intake may differ by tumor hormone receptor status 12, 13, 14, 15, 16, 17.

To date, most studies on fiber intake and breast cancer risk have been conducted in non‐Hispanic White (NHW) women. Data on Hispanic and African American women are sparse, although fiber intake and fiber sources have been shown to vary substantially across racial/ethnic populations in the United States (U.S.) 18, 19, 20, 21, 22. Hispanics, for example, have a higher fiber intake than African Americans and NHWs 18, 19, 20, 21, 22, and they consume fiber from different sources, with a higher proportion of fiber from beans 20, 21, 22, particularly among foreign‐born Hispanics 23.

We examined the relation between intake of fiber and fiber‐rich foods and breast cancer risk in a population‐based case–control study conducted in a multiethnic population with a wide range of dietary fiber intake from diverse sources.

## Materials and Methods

### Study population

Women included in this study were participants in the San Francisco Bay Area Breast Cancer Study, a population‐based case–control study conducted from 1995 to 2004 24, 25. Through the Greater Bay Area Cancer Registry, we identified 17,581 women aged 35–79 years and newly diagnosed with a first primary invasive breast cancer, including African Americans and NHWs diagnosed between 1 April 1995 and 30 April 1999 and Hispanics diagnosed between 1 April 1995 and 30 April 2002. A brief telephone screening interview assessed study eligibility and self‐reported race/ethnicity. Of those screened (89% participation), all Hispanics and African Americans and a 10% random sample of NHWs were selected into the study (*n *= 2571). Of those selected, 2258 (88%) completed an in‐person interview, including 1119 (89%) Hispanic, 543 (87%) African American, and 596 (86%) NHW cases.

Controls were identified through random‐digit dialing 24. From the pool of potentially eligible controls, 3771 were randomly selected and frequency matched to cases according to the expected race/ethnicity and 5‐year age distribution of cases. Telephone screening (92% participation) identified 3170 control women who met the study eligibility criteria. Of these, 2706 (85%) completed the in‐person interview, including 1462 (88%) Hispanics, 598 (82%) African Americans, and 646 (83%) NHWs.

The study was approved by the Institutional Review Board of the Cancer Prevention Institute of California (Fremont, California, U.S.).

### Data collection

A structured questionnaire in English or Spanish was administered by trained bilingual and bicultural interviewers at the participants’ home, collecting information on demographic background, lifestyle factors, menstrual and reproductive history, hormone use, and medical history up to the reference year (defined as the calendar year before diagnosis for cases or before selection into the study for controls). Measurements of weight and height were taken during the interview. Usual dietary intake (frequency of consumption and portion size) in the reference year was assessed using a food frequency questionnaire (FFQ) adapted from the 1995 106‐item Block Health History and Habits Questionnaire, with the addition of food items commonly consumed by Hispanic and African‐American women in California, including a variety of beans 26. With regard to fiber sources, the FFQ asked about consumption of single or groups of *fruits* (apples and apple sauce; bananas and plantains; oranges, tangerines, and grapefruits; cantaloupe; prunes; and berries such as blueberries, blackberries, strawberries, and raspberries), *vegetables* (soups with tomatoes or carrots; fresh or stewed tomatoes, and salsa; raw or cooked carrots; bell peppers and chile rellenos; broccoli; cooked spinach, mustard greens, turnip greens, collards, kale, and chard; alfalfa sprouts; regular bean sprouts; and lettuce), *beans* (garbanzo beans; other beans such as pinto kidney, black, red, lima, refried, peas, and black‐eyed peas, including beans in burritos or other dishes; and frijoles de olla), and *grains* (rice; white bread; dark bread; noodles and pasta; cheese dishes without tomato sauce such as mac and cheese or quesadillas; fiber or bran cereals; other cold cereals; cooked cereals such as oatmeal, cream of wheat, and grits; flour tortillas; corn tortillas and cornbread; rolls, buns, bagels, and English muffins; biscuits and muffins; pancakes, waffles, and French toast; doughnuts, pastries, churros, and pan dulce; cakes and cookies; and salty snacks). The same FFQ was administered to women of all races/ethnicities. Daily intake of specific nutrients was estimated using the DIETSYS software that linked the FFQ data to a nutrient database, which was adapted from nutrient databases developed for the Block 1995 FFQ, and the FFQ used for the Study of Women's Health Across the Nation 27. Data on estrogen receptor (ER) and progesterone receptor (PR) status were obtained from the cancer registry.

### Study variables

We examined intake (g/day) of total fiber; fiber from specific foods (beans, grains, vegetables, and fruits); and fiber‐rich foods (total beans, total grains, total vegetables, and total fruits). Body mass index (BMI, kg/m^2^) was calculated as self‐reported weight (kg) in the reference year divided by measured squared height (m). For the small number of participants who did not report their weight in the reference year (1% of cases and 2% of controls), we used measured weight, and for those who declined the height measurement (7% of cases and 7% of controls), we used self‐reported height for the BMI calculation. We used self‐reported instead of measured weight because of concern about treatment‐related and disease‐related weight gain after diagnosis. Average lifetime physical activity (hours/week) between menarche and the reference year was estimated from self‐reported histories of exercise, walking and bicycling, strenuous indoor and outdoor chores, and occupational activity 24. Menopausal status was determined using methods defined previously 24, 25.

### Statistical analysis

Of the 4964 women who completed the in‐person interview, we excluded 69 cases and 63 controls with total daily energy intake <600 kcal or >5000 kcal (indicative of unreliable dietary recall) and 54 cases and 72 controls with missing covariate data, leaving 2135 cases and 2571 controls for the analysis. Using multivariable unconditional logistic regression, we estimated odds ratios (OR) and 95% confidence intervals (CI) for breast cancer risk associated with intake of total fiber, intake of fiber from specific foods (beans, grains, and vegetables and fruits combined), and intake of fiber‐rich foods (beans, grains, vegetables, and fruits). Models were adjusted for total energy intake using the residual method (with logarithm transformation of nutrients and energy intake) 28. Quartiles or tertiles of energy‐adjusted nutrient or food intake were determined according to the distributions among all controls combined. Tests of trend were conducted by treating the quartiles or tertiles as ordered four‐category or three‐category variables, respectively.

The multivariable models were adjusted for age (continuous), race/ethnicity and birthplace (foreign‐born Hispanics, U.S.‐born Hispanics, African Americans, and NHWs), and variables associated with breast cancer risk (Table 1), including education (some high school or less, high school or vocational/technical school graduate, some college, and college graduate), first‐degree family history of breast cancer (no, yes), personal history of biopsy‐confirmed benign breast disease (no, yes), age at menarche (<14 years, ≥14 years), menopausal status and hormone therapy (HT) use (premenopausal, postmenopausal and never or past HT use, postmenopausal and current HT use, and unknown menopausal status or HT use), parity and age at first full‐term pregnancy (FFP) (nulliparous, parity 1–2 and FFP <30 years, parity 1–2 and FFP ≥30 years, parity ≥3 and FFP <25 years, or parity ≥3 and FFP ≥25 years), lifetime breastfeeding (none, <12, ≥12 months), average lifetime physical activity (hours/week, quartiles), BMI (<25.0, 25.0–29.9, ≥30.0 kg/m^2^), daily alcohol consumption (none, <5, 5–9, ≥10 g), daily energy intake (continuous), and daily fat intake (energy‐adjusted, quartiles). We stratified the analyses by race/ethnicity and birthplace and by hormone receptor status. Polytomous logistic regression was used to estimate the associations between the dietary variables and risk of hormone receptor‐positive (ER+ and/or PR+) and ER‐PR‐ breast cancer. Analyses for ER+ and/or PR+ breast cancer were adjusted for the same variables as for overall breast cancer. Analyses for ER‐PR‐ breast cancer were adjusted for age, race/ethnicity and birthplace, age at menarche, menopausal status and HT use, breastfeeding, physical activity, and energy and fat intake. All tests of significance were two‐sided with *P *< 0.05 as the significant cutoff point. Analyses were performed using SAS Version 9.4 (SAS Institute, Cary, NC).

**Table 1:** Characteristics of cases and controls, San Francisco Bay Area Breast Cancer Study

	Cases (*n *= 2135)		Controls (*n *= 2571)		*P* value^b^
	*n*	%^a^	*n*	%^a^	
Age (years)					
35–44	400	19	508	20	
45–54	637	30	771	30	
55–64	533	25	637	25	
65–74	406	19	506	20	
≥75	159	8	149	6	
Race/ethnicity and birthplace					
Foreign‐born Hispanics	529	25	931	36	
U.S.‐born Hispanics	541	25	460	18	
African Americans	493	23	557	22	
Non‐Hispanic Whites	572	27	623	24	
Estrogen receptor (ER) and progesterone receptor (PR) status					
ER+ and/or PR+	1498	70			
ER‐PR‐	414	19			
Unknown	223	10			
Education					
Some high school or less	547	26	911	35	<0.01
High school or vocational/technical school graduate	581	27	672	26	
Some college	536	25	512	20	
College graduate	471	22	476	19	
Family history of breast cancer in first‐degree relatives					
No	1797	84	2287	89	<0.01
Yes	338	16	284	11	
Personal history of biopsy‐confirmed benign breast disease					
No	1692	79	2188	85	<0.01
Yes	443	21	383	15	
Age at menarche (years)					
<12	517	24	570	22	<0.01
12–13	1093	51	1252	49	
≥14	525	25	749	29	
Parity					
Nulliparous	330	16	279	11	<0.01
Parous	1805	85	2292	89	
Number of full‐term pregnancies, parous women					
1	311	17	320	14	<0.01
2	565	31	586	26	
3	422	23	567	25	
≥4	507	28	819	36	
Age at first full‐term pregnancy (years), parous women					
<20	599	33	881	38	0.01
20–24	548	30	639	28	
25–29	445	25	532	23	
≥30	213	12	240	11	
Lifetime breastfeeding (months), parous women					
0	776	43	804	35	<0.01
<12	553	31	657	29	
≥12	476	26	831	36	
Menopausal status					
Premenopausal	671	31	806	31	0.58
Postmenopausal	1310	61	1559	61	
Unknown	154	7	206	8	
Hormone therapy use, postmenopausal women					
Never	515	39	638	41	<0.01
Former	283	22	689	44	
Current	496	38	214	14	
Unknown	16	1	18	1	
Lifetime physical activity (quartiles, hours/week)^c^					
Q1: < 6.8	598	28	638	25	<0.01
Q2: 6.8–14.1	594	28	643	25	
Q3: 14.2–25.1	475	22	648	25	
Q4: ≥25.2	468	22	642	25	
BMI (kg/m^2^), premenopausal women					
<25.0	297	44	262	33	<0.01
25.0–29.9	195	29	274	34	
30.0	179	27	270	34	
BMI (kg/m^2^), postmenopausal women					
<25.0	408	31	412	26	0.02
25.0–29.9	434	33	550	35	
≥30.0	468	36	597	38	
Alcohol consumption^d^ (g/day)					
0	1139	53	1530	60	<0.01
0.1–4.9	486	23	553	22	
5.0–9.9	130	6	154	6	
≥10	380	18	334	13	
Total daily energy intake (quartiles, kcal)^c^^,^ d ^,^ e					
Q1: < 1432	520	24	657	26	<0.01
Q2: 1432–1903	539	25	638	25	
Q3: 1904–2538	546	26	629	24	
Q4: ≥2539	530	25	647	25	
Energy‐adjusted daily fat intake (quartiles, g)^c^^,^ d					
Q1: < 56.0	404	19	645	25	<0.01
Q2: 56.0–65.9	454	21	643	25	
Q3: 66.0–76.7	586	27	639	25	
Q4: ≥76.8	691	32	644	25	

a Percentages may not add up to 100% due to rounding.

b From Chi‐square test.

c Quartiles among all controls.

d In reference year (calendar year before diagnosis for cases or before selection into the study for controls).

e Excluding energy from alcohol.

## Results

Compared with controls, cases had higher education, earlier menarche, older age at first full‐term pregnancy, shorter duration of breastfeeding, lower physical activity, lower BMI, higher alcohol consumption, and higher caloric and fat intake; higher proportions of cases than controls had a family history of breast cancer or a personal history of benign breast disease and were nulliparous or current HT users (Table 1).

Control women had a higher fiber intake and consumed a higher percentage of fiber from beans than cases (Table 2). Foreign‐born Hispanic controls on average had the highest fiber intake, and the differences in fiber intake between foreign‐born Hispanics and other groups remained statistically significant after normalizing by energy intake (data not shown). Among foreign‐born Hispanics, the largest proportion of fiber came from beans, whereas among U.S.‐born Hispanics, African Americans, and NHWs, vegetables and fruits were the primary source of fiber intake. Large proportions of controls did not meet the recommendations for daily fiber intake (25 g/day for women ages ≤50 years, 22 g/day for women ages >50 years) 29, ranging from 45 to 92%. Adherence to recommended daily fiber intake was highest for foreign‐born Hispanics and lowest for African Americans.

**Table 2:** Dietary intake among controls, by race/ethnicity and birthplace a, San Francisco Bay Area Breast Cancer Study

	Cases (*n *= 2135)	Controls				
		All (*n *= 2571)	Foreign‐born Hispanics (*n *= 931)	U.S.‐born Hispanics (*n *= 460)	African Americans (*n *= 557)	non‐Hispanic Whites (*n *= 623)
Total fiber, g/day^b^^,^ c	21.3 ± 0.3	23.6 ± 0.3	31.1 ± 0.5	22.1 ± 0.5	17.1 ± 0.4	18.6 ± 0.4
% of fiber from beans^b^^,^ c	18.8 ± 0.4	22.4 ± 0.4	37.5 ± 0.7	23.1 ± 0.8	10.2 ± 0.6	9.7 ± 0.5
% of fiber from grains^b^^,^ c	36.3 ± 0.3	34.6 ± 0.3	28.3 ± 0.4	33.9 ± 0.6	40.3 ± 0.7	39.7 ± 0.6
% of fiber from vegetables and fruits^b^^,^ c	40.3 ± 0.4	38.4 ± 0.3	30.9 ± 0.5	35.5 ± 0.7	44.7 ± 0.7	45.8 ± 0.6
Total beans, g/day^b^^,^ c	69.0 ± 2.6	90.7 ± 2.4	168.8 ± 5.3	78.2 ± 4.3	32.0 ± 2.5	32.3 ± 2.4
Total grains, g/day^b^^,^ c	333.8 ± 3.9	353.3 ± 3.6	412.5 ± 6.3	331.5 ± 7.8	314.7 ± 8.1	317.5 ± 6.5
Total vegetables and fruit, g/day^b^^,^ c	422.0 ± 5.9	438.9 ± 5.4	501.3 ± 9.9	381.0 ± 10.8	401.4 ± 12.3	417.2 ± 9.8
Total vegetables, g/day^c^	213.4 ± 3.4	221.5 ± 3.0	255.0 ± 5.3	194.9 ± 6.2	191.0 ± 9.6	217.0 ± 6.1
Total fruits, g/day^c^	208.6 ± 3.8	217.4 ± 3.4	246.2 ± 6.5	186.1 ± 6.7	210.4 ± 8.6	200.2 ± 5.6
% of women ages ≤50 years meeting guidelines for total fiber intake^b^^,^ c ^,^ d	20.3	27.4	51.1	12.0	8.3	11.1
% of women ages >50 years meeting guidelines for total fiber intake^b^^,^ c ^,^ d	22.6	31.1	54.6	31.2	13.2	17.9

a Values in the table are age‐adjusted least‐square means ± standard error or otherwise specified.

b *P* < 0.05 when comparing cases and controls.

c *P* < 0.05 when comparing the three racial/ethnic control groups (Hispanic, African American, and non‐Hispanic Whites) and *P* < 0.05 between foreign‐born and U.S.‐born Hispanic controls.

d U.S. Department of Agriculture and U.S. Department of Health and Human Services 2010 guidelines for total fiber intake are 25 g/day for women ages ≤50 years and 22 g/day for women >50 years.

Associations between breast cancer risk and intake of fiber, different sources of fiber, and fiber‐rich foods are shown in Table 3. Total fiber intake was associated with reduced breast cancer risk (high vs. low quartile: OR = 0.75, 95% CI = 0.60–0.93), but there was no linear trend of decreasing risk with increasing intake (p‐trend = 0.07). Statistically significant inverse trends emerged only when we considered intake of fiber from specific sources or intake of fiber‐rich foods. Reduced risk was associated with high intake (high vs. low quartile) of bean fiber (OR = 0.79, 95% CI = 0.63–0.98, p‐trend = 0.01), total beans (OR = 0.81, 95% CI = 0.66–1.01, p‐trend = 0.03), and total grains (OR = 0.82, 95% CI = 0.68–0.99, p‐trend  = 0.05). No associations were found for fiber from grains or fiber from vegetables and fruits combined, or for intake of total vegetables and/or fruits. Findings were similar for premenopausal and postmenopausal women (data not shown).

**Table 3:** Breast cancer risk and intake of energy‐adjusted fiber, sources of fiber, and fiber‐rich food groups, San Francisco Bay Area Breast Cancer Study

	Energy‐adjusted intake					*P*‐trend
	(continuous^a^)	(quartiles, g/day)				
Total fiber intake		Q1: <15.2	Q2: 15.2–19.8	Q3: 19.9–26.5	Q4: >26.5	
No. of cases	2135	702	503	551	379	
No. of controls	2571	636	649	641	645	
OR (95% CI)^b^	0.93 (0.85–1.01)	1.0	0.69 (0.58–0.82)	0.90 (0.75–1.08)	0.75 (0.60–0.93)	0.07
Fiber from beans		Q1: <1.83	Q2: 1.83–4.17	Q3: 4.18–9.88	Q4: >9.88	
No. of cases	2135	678	560	528	369	
No. of controls	2571	646	591	692	642	
OR (95% CI)^b^	0.93 (0.84–1.04)	1.0	0.90 (0.76–1.07)	0.78 (0.65–0.93)	0.79 (0.63–0.98)	0.01
Fiber from grains		Q1: <5.88	Q2: 5.88–7.47	Q3: 7.48–9.51	Q4: >9.51	
No. of cases	2135	612	513	501	509	
No. of controls	2571	644	642	644	641	
OR (95% CI)^b^	0.92 (0.75–1.14)	1.0	0.89 (0.75–1.06)	0.92 (0.76–1.09)	0.95 (0.80–1.14)	0.63
Fiber from vegetables and fruits		Q1: <6.19	Q2: 6.19–8.05	Q3: 8.06–10.79	Q4: >10.79	
No. of cases	2135	578	509	529	519	
No. of controls	2571	643	642	637	649	
OR (95% CI)^b^	0.96 (0.81–1.14)	1.0	0.88 (0.75–1.05)	0.91 (0.77–1.08)	0.95 (0.80–1.14)	0.66
Total beans		Q1: <6.4	Q2: 6.4–43.0	Q3: 43.1–109.6	Q4: >109.6	
No. of cases	2135	638	632	497	368	
No. of controls	2571	645	641	642	643	
OR (95% CI)^b^	0.97 (0.89–1.05)	1.0	0.99 (0.83–1.17)	0.84 (0.70–1.02)	0.81 (0.66–1.01)	0.03
Total grains		Q1: <246.2	Q2: 246.2–312.2	Q3: 312.3–391.3	Q4: >391.3	
No. of cases	2135	617	588	534	396	
No. of controls	2571	642	644	643	642	
OR (95% CI)^b^	0.92 (0.87–0.97)	1.0	1.05 (0.89–1.24)	1.01 (0.85–1.20)	0.82 (0.68–0.99)	0.05
Total vegetables and fruits		Q1: <266.3	Q2: 266.3–385.8	Q3: 385.9–545.4	Q4: >545.4	
No. of cases	2135	617	472	545	501	
No. of controls	2571	642	644	644	641	
OR (95% CI)^b^	1.00 (0.97–1.02)	1.0	0.80 (0.68–0.95)	0.93 (0.78–1.10)	0.95 (0.79–1.13)	0.86
Total vegetables		Q1: <118.2	Q2: 118.2–188.6	Q3: 188.7–277.3	Q4: >277.3	
No. of cases	2135	571	529	523	512	
No. of controls	2571	643	646	640	642	
OR (95% CI)^b^	0.99 (0.95–1.03)	1.0	0.98 (0.82–1.16)	0.98 (0.82–1.16)	1.01 (0.84–1.20)	0.96
Total fruits		Q1: <106.9	Q2: 106.9–178.0	Q3: 178.1–279.9	Q4: >279.9	
No. of cases	2135	581	498	525	531	
No. of controls	2571	641	645	641	644	
OR (95% CI)^b^	1.00 (0.96–1.04)	1.0	0.88 (0.74–1.04)	0.98 (0.82–1.16)	1.03 (0.86–1.23)	0.53

a Per 10 g/day for total fiber, fiber from beans, fiber from grains, and fiber from vegetables and fruits; per 100 g/day for total beans, otal grains, and total vegetables and/or fruits.

b Adjusted for age, race/ethnicity and birthplace, education, history of breast cancer in first‐degree relatives, personal history of benign breast disease, age at menarche, parity and age at first full‐term pregnancy, breastfeeding, menopausal status and hormone therapy use, average lifetime physical activity, current BMI, alcohol consumption, total energy intake (continuous, excluding energy from alcohol), and energy‐adjusted daily fat intake.

For hormone receptor‐positive breast cancer (Table 4), there was a borderline inverse trend for total fiber intake (p‐trend = 0.06), with significant risk reductions for women in the 2nd and 4th quartile of total fiber intake (OR = 0.66, 95% CI = 0.55–0.80, and OR = 0.72, 95% CI = 0.57–0.92, respectively). Similarly for bean fiber, the inverse trend was of borderline significance (p‐trend = 0.05). Strong inverse associations and linear trends were found for ER‐PR‐ disease; reduced risks were associated with high intake (high vs. low quartile) of bean fiber (OR = 0.66, 95% CI = 0.45–0.97, p‐trend = 0.02), total beans (OR = 0.72, 95% CI = 0.50–1.05, p‐trend = 0.04), and total grains (OR = 0.64, 95% CI = 0.46–0.90, p‐trend = 0.01). For bean fiber and total beans, inverse associations with high intake (high vs. low tertile) were found among foreign‐born Hispanics only (OR = 0.43, 95% CI = 0.24–0.80, p‐trend = 0.01 and OR = 0.49, 95% CI = 0.25–0.93, p‐trend = 0.02, respectively), whereas for total grains, reduced risk was found among NHW women only (OR = 0.39, 95% CI = 0.17–0.89, p‐trend = 0.04; OR per 100 g/day: 0.64, 95% CI = 0.48–0.85) (Table 5).

**Table 4:** Breast cancer risk and intake of energy‐adjusted fiber, sources of fiber, and fiber‐rich food groups, by ER/PR status, San Francisco Bay Area Breast Cancer Study

	Energy‐adjusted intake					*P*‐trend
	(continuous^a^)	(quartiles, g/day)				
Total fiber intake		Q1: <15.2	Q2: 15.2–19.8	Q3: 19.9–26.5	Q4: >26.5	
No. of controls	2571	636	649	641	645	
No. of ER+ and/or PR+ cases	1498	490	348	395	265	
OR (95% CI)^b^	0.92 (0.83–1.01)	1.0	0.66 (0.55–0.80)	0.88 (0.72–1.08)	0.72 (0.57–0.92)	0.06
No. of ER‐PR‐ cases	414	143	87	106	78	
OR (95% CI)^c^	0.96 (0.82–1.12)	1.0	0.65 (0.48–0.87)	0.93 (0.68–1.27)	0.81 (0.55–1.18)	0.53
Fiber from beans		Q1: <1.83	Q2: 1.83–4.17	Q3: 4.18–9.88	Q4: >9.88	
No. of controls	2571	646	591	692	642	
No. of ER+ and/or PR+ cases	1498	472	398	366	262	
OR (95% CI)^b^	0.92 (0.81–1.04)	1.0	0.91 (0.75–1.10)	0.79 (0.64–0.96)	0.84 (0.66–1.06)	0.05
No. of ER‐PR‐ cases	414	132	103	106	73	
OR (95% CI)^c^	0.95 (0.79–1.15)	1.0	0.86 (0.63–1.15)	0.73 (0.53–0.99)	0.66 (0.45–0.97)	0.02
Fiber from grains		Q1: <5.88	Q2: 5.88–7.47	Q3: 7.48–9.51	Q4: >9.51	
No. of controls	2571	644	642	644	641	
No. of ER+ and/or PR+ cases	1498	412	361	364	361	
OR (95% CI)^b^	0.92 (0.72–1.16)	1.0	0.92 (0.77–1.11)	0.98 (0.81–1.19)	0.97 (0.79–1.18)	0.88
No. of ER‐PR‐ cases	414	122	107	95	90	
OR (95% CI)^c^	0.93 (0.64–1.35)	1.0	0.94 (0.71–1.26)	0.91 (0.67–1.23)	0.92 (0.67–1.26)	0.57
Fiber from vegetables and fruits		Q1: <6.19	Q2: 6.19–8.05	Q3: 8.06–10.79	Q4: >10.79	
No. of controls	2571	643	642	637	649	
No. of ER+ and/or PR+ cases	1498	386	359	383	370	
OR (95% CI)^b^	0.97 (0.89–1.07)	1.0	0.91 (0.75–1.10)	0.94 (0.78–1.14)	0.95 (0.78–1.17)	0.73
No. of ER‐PR‐ cases	414	139	86	87	102	
OR (95% CI)^c^	1.01 (0.75–1.35)	1.0	0.67 (0.50–0.91)	0.73 (0.54–0.99)	0.97 (0.72–1.30)	0.73
Total beans		Q1: <6.4	Q2: 6.4–43.0	Q3: 43.1–109.6	Q4: >109.6	
No. of controls	2571	645	641	642	643	
No. of ER+ and/or PR+ cases	1498	444	444	353	257	
OR (95% CI)^b^	0.97 (0.89–1.07)	1.0	0.99 (0.82–1.19)	0.87 (0.71–1.06)	0.84 (0.67–1.07)	0.09
No. of ER‐PR‐ cases	414	125	120	95	74	
OR (95% CI)^c^	0.96 (0.83–1.11)	1.0	0.93 (0.69–1.24)	0.74 (0.54–1.03)	0.72 (0.50–1.05)	0.04
Total grains		Q1: <246.2	Q2: 246.2–312.2	Q3: 312.3–391.3	Q4: >391.3	
No. of controls	2571	642	644	643	642	
No. of ER+ and/or PR+ cases	1498	423	408	374	293	
OR (95% CI)^b^	0.94 (0.88–1.00)	1.0	1.09 (0.91–1.32)	1.03 (0.85–1.25)	0.89 (0.73–1.10)	0.28
No. of ER‐PR‐ cases	414	129	117	100	68	
OR (95% CI)^c^	0.87 (0.78–0.96)	1.0	0.95 (0.72–1.26)	0.88 (0.65–1.18)	0.64 (0.46–0.90)	0.01
Total vegetables and fruits		Q1: <266.3	Q2: 266.3–385.8	Q3: 385.9–545.4	Q4: >545.4	
No. of controls	2571	642	644	644	641	
No. of ER+ and/or PR+ cases	1498	417	337	377	367	
OR (95% CI)^b^	1.00 (0.97–1.03)	1.0	0.83 (0.68–1.00)	0.91 (0.75–1.10)	0.97 (0.80–1.19)	0.96
No. of ER‐PR‐ cases	414	132	96	94	92	
OR (95% CI)^c^	1.00 (0.95–1.05)	1.0	0.82 (0.61–1.10)	0.86 (0.64–1.15)	0.94 (0.69–1.28)	0.70
Total vegetables		Q1: <118.2	Q2: 118.2–188.6	Q3: 188.7–277.3	Q4: >277.3	
No. of controls	2571	643	646	640	642	
No. of ER+ and/or PR+ cases	1498	390	374	374	360	
OR (95% CI) b	0.98 (0.94–1.04)	1.0	0.98 (0.81–1.18)	0.99 (0.82–1.20)	0.98 (0.81–1.20)	0.90
No. of ER‐PR‐ cases	414	119	103	101	91	
OR (95% CI)^c^	1.01 (0.93–1.09)	1.0	0.99 (0.74–1.33)	1.02 (0.76–1.37)	1.00 (0.73–1.36)	0.96
Total fruits		Q1: <106.9	Q2: 106.9–178.0	Q3: 178.1–279.9	Q4: >279.9	
No. of controls	2571	641	645	641	644	
No. of ER+ and/or PR+ cases	1498	398	342	362	396	
OR (95% CI)^b^	1.01 (0.97–1.06)	1.0	0.87 (0.71–1.05)	0.95 (0.78–1.15)	1.08 (0.88–1.31)	0.35
No. of ER‐PR‐ cases	414	128	99	93	94	
OR (95% CI)^c^	0.99 (0.92–1.06)	1.0	0.85 (0.63–1.14)	0.89 (0.66–1.20)	0.96 (0.71–1.32)	0.84

a Per 10 g/day for total fiber, fiber from beans, fiber from grains, and fiber from vegetables and fruits; per 100 g/day for total beans, otal grains, and total vegetables and/or fruits.

b Adjusted for age, race/ethnicity and birthplace, education, history of breast cancer in first‐degree relatives, personal history of benign breast disease, age at menarche, parity and age at first full‐term pregnancy, breastfeeding, menopausal status and menopausal hormone therapy use, average lifetime physical activity, current BMI, alcohol consumption, total energy intake (continuous, excluding energy from alcohol), and energy‐adjusted daily fat intake.

c Adjusted for age, race/ethnicity and birthplace, age at menarche, breastfeeding, menopausal status and hormone therapy use, average lifetime physical activity, total energy intake (continuous, excluding energy from alcohol), and energy‐adjusted daily fat intake.

**Table 5:** Risk of ER‐PR‐ breast cancer risk and intake of energy‐adjusted fiber from beans, total beans, and total grains, by race/ethnicity and birthplace

	Energy‐adjusted intake				*P*‐trend
	(continuous^a^)	(tertiles, g/day)			
Fiber from beans		T1: <2.41	T2: 2.41–7.52	T3: >7.52	
Foreign‐born Hispanics					
No. of cases	118	20	38	60	
No. of controls	931	67	256	608	
OR (95% CI)^b^	0.87 (0.67–1.13)	1.0	0.55 (0.29–1.02)	0.43 (0.24–0.80)	0.01
U.S.‐born Hispanics					
No. of cases	109	26	53	30	
No. of controls	460	100	226	134	
OR (95% CI)^b^	1.14 (0.77–1.67)	1.0	1.02 (0.59–1.75)	1.07 (0.57–2.02)	0.79
African Americans					
No. of cases	122	82	34	6	
No. of controls	557	318	186	53	
OR (95% CI)^b^	0.99 (0.55–1.75)	1.0	0.76 (0.48–1.19)	0.56 (0.23–1.38)	0.12
Non‐Hispanic Whites					
No. of cases	65	39	21	5	
No. of controls	623	365	204	54	
OR (95% CI)^b^	1.02 (0.47–2.20)	1.0	0.93 (0.52–1.66)	1.03 (0.37–2.87)	0.94
Total beans		T1: <14.7	T2: 14.7–80.5	T3: >80.5	
Foreign‐born Hispanics					
No. of cases	118	17	46	55	
No. of controls	931	64	289	578	
OR (95% CI)^b^	0.87 (0.70–1.09)	1.0	0.67 (0.35–1.27)	0.49 (0.25–0.93)	0.02
U.S.‐born Hispanics					
No. of cases	109	27	54	28	
No. of controls	460	87	235	138	
OR (95% CI)^b^	1.05 (0.78–1.41)	1.0	0.82 (0.48–1.42)	0.80 (0.42–1.52)	0.53
African Americans					
No. of cases	122	80	31	11	
No. of controls	557	330	161	66	
OR (95% CI)^b^	1.14 (0.83–1.58)	1.0	0.77 (0.48–1.23)	0.90 (0.44–1.83)	0.45
Non‐Hispanic Whites					
No. of cases	65	39	20	6	
No. of controls	623	368	188	67	
OR (95% CI)^b^	0.99 (0.60–1.65)	1.0	1.07 (0.59–1.92)	1.02 (0.40–2.63)	0.87
Total grains		T1: <268.7	T2: 268.7–361.9	T3: >361.9	
Foreign‐born Hispanics					
No. of cases	118	26	46	46	
No. of controls	931	192	335	404	
OR (95% CI)^b^	0.93 (0.77–1.13)	1.0	1.13 (0.66–1.91)	1.05 (0.61–1.81)	0.91
U.S.‐born Hispanics					
No. of cases	109	42	49	18	
No. of controls	460	179	162	119	
OR (95% CI)^b^	0.92 (0.75–1.14)	1.0	1.44 (0.89–2.34)	0.71 (0.38–1.33)	0.53
African Americans					
No. of cases	122	63	29	30	
No. of controls	557	236	174	147	
OR (95% CI)^b^	0.86 (0.71–1.03)	1.0	0.64 (0.39–1.05)	0.82 (0.48–1.39)	0.30
Non‐Hispanic Whites					
No. of cases	65	29	26	10	
No. of controls	623	242	203	178	
OR (95% CI)^b^	0.64 (0.48–0.85)	1.0	0.96 (0.53–1.74)	0.39 (0.17–0.89)	0.04

a Per 10 g/day for fiber from beans and per 100 g/day for total beans and total grains.

b Adjusted for age, race/ethnicity and birthplace, age at menarche, breastfeeding, menopausal status and hormone therapy use, average lifetime physical activity, total energy intake (continuous, excluding energy from alcohol), and energy‐adjusted daily fat intake.

## Discussion

In this population‐based case–control study, fiber intake was most strongly associated with risk of ER‐PR‐ breast cancer. Inverse trends were associated with intake of bean fiber (p‐trend = 0.02), total beans (p‐trend = 0.04), and total grains (p‐trend = 0.01), with risk reductions associated with high intake ranging from 28 to 36%. For hormone receptor‐positive breast cancer, inverse trends for total fiber and bean fiber were of borderline significance.

We found a 25% reduction in breast cancer risk associated with high versus low total fiber intake and no association with fiber from vegetables and fruits combined or total intake of vegetables and/or fruits. A recent meta‐analysis of 16 prospective studies reported that high intake of total fiber 4, fruits 30, and fruits and vegetables combined 30 were associated with reduced breast cancer risk, but no association was found for fruit fiber or vegetable fiber 4, whereas some studies reported inverse associations with fiber from vegetables and/or fruits 7, 8, 12, 13, 31, 32.

In our study, the strongest inverse associations were found for bean fiber and total bean intake, but limited to ER‐PR‐ breast cancer and foreign‐born Hispanic women. Few studies have assessed the relation between breast cancer and intake of bean fiber or total beans, possibly due to its minor role as a source of fiber in most NHW populations. Of the four studies that reported on the association with fiber from beans or legumes, only the Nurses’ Health Study II found an association, albeit marginally significant, with breast cancer risk overall 10, 15, 17, 33 and no prior study has shown heterogeneity by ER/PR status 15, 17. In our study population, foreign‐born Hispanic controls had the highest mean fiber intake, and beans were their main source of fiber, accounting for 38% of total fiber intake (compared to 23% in U.S.‐born Hispanics, 10% in NHWs, and 9% in African Americans). The large number of foreign‐born Hispanics in our study greatly widened the range of fiber exposure from beans and may be the reason why we were able to detect an inverse association with bean fiber among foreign‐born Hispanics only. In the meta‐analysis by Aune et al. 4, the inverse association with total fiber intake was only observed in studies with a wide range of fiber intake or high levels of intake (>25 g/day). In our study, more than half foreign‐born Hispanics met the fiber intake recommendations for their age (25 g/day for ages ≤50 years and 22 g/day for women >50 years). These intake levels were much higher than intake levels of African Americans and NHWs. Thus, it may not be possible to detect inverse associations with fiber in populations with a low or narrow range of fiber intake 4.

Data on fiber intake and breast cancer risk in Hispanic populations are very limited. A small study from Mexico (68 cases, 69 controls) reported a marginal inverse trend (*P *= 0.08) for total fiber intake in premenopausal women 34. In a study from Uruguay (351 cases, 356 hospital controls), higher total fiber intake was associated with a 49% lower risk of breast cancer, independent of type of fiber (soluble vs. insoluble) or menopausal status, and significant inverse trends were found for fiber from grains and fiber from vegetables, but not for fiber from fruits 8. Fiber from beans was not assessed in that study.

Data on the association with fiber from grains are inconsistent. While some studies have reported inverse trends with fiber from grains 6, 8, 9, 11, 35, 36, a recent meta‐analysis found no association with cereal fiber 4. We found no association with fiber from grains, but total grain intake was associated with reduced risk of ER‐PR‐ breast cancer, particularly among NHW women. Although recent studies in NHWs have shown inverse associations between intake of grains and breast cancer risk 36, 37, some studies found no differences by hormone receptor status 35, 38.

In our study, inverse associations with bean fiber, total beans, and total grains were strongest for ER‐PR‐ breast cancer. Only a few previous studies examined the relation between fiber intake and breast cancer subtypes. Some studies also found inverse associations limited to ER‐PR‐ 14, 15, 17 or ER‐ 16 disease, other studies reported inverse associations with fiber intake for ER+ and/or PR+ tumors 13, positive associations for ER+PR+ disease 17, or no differences by ER/PR status 12. Many of the currently known breast cancer risk factors are more strongly associated with hormone receptor‐positive disease; few risk factors have been identified for ER‐PR‐ or ER‐ breast cancer subtypes 39, 40, 41. In this context, our finding that high intake of bean fiber, beans, and grains is associated with reduced risk of ER‐PR‐ breast cancer warrants further investigation in studies with larger numbers of ER‐PR‐ cases. Except for alcohol consumption, the role of dietary factors in breast cancer etiology remains inconclusive 42, 43. There is emerging evidence, however, that associations with some dietary factors or dietary patterns may differ by tumor hormone receptor status. In addition to fiber intake 15, 17, intake of vegetable fiber 14 and diets rich in fruits and vegetables 44, 45, 46, 47, 48, 49 have also been inversely associated with risk of ER‐PR‐ or ER‐ disease, whereas a high intake of total fat and saturated fat has been associated with increased risk of ER+PR+ breast cancer, but not with hormone receptor‐negative disease 50.

The mechanisms underlying the possible inverse association with fiber intake are not yet clearly understood. Inverse associations with ER‐PR‐ breast cancer suggest the importance of non‐estrogen‐mediated mechanisms (e.g., insulin growth factor pathway) 51, 52. Differences in associations with different sources of fiber may reflect different effects from soluble versus insoluble fiber 4. Some early studies suggested that soluble and insoluble fiber function differently in inhibiting enterohepatic circulation of estrogens, thereby lowering circulating estrogen levels, one of the main mechanisms believed to underlie the protective effect of fiber on breast cancer risk 53, 54, 55. Soluble fiber, which is the major fraction of bean fiber, has been found to be more effective in slowing glucose absorption, reducing insulin secretion, and regulating the bioavailability of insulin‐like growth factors 51, 56, another important pathway in breast cancer etiology 51, 52. The meta‐analysis by Aune et al. 4, however, found similar results for soluble fiber [summary relative risk (RR)  = 0.91, 95% CI = 0.84–0.99] and insoluble fiber (summary RR =  0.96, 95% CI = 0.88–1.04) 4.

Phytoestrogens are unlikely to account for the associations observed for bean fiber, as the types of beans commonly consumed by our study participants (e.g., pinto, garbanzo, and kidney beans by Hispanics and NHWs versus pinto beans, black‐eyed peas, and small white beans by African Americans) are generally not rich sources of lignans or isoflavones 57, 58, except for garbanzo beans, which are rich in biochanin A, a minor isoflavone. Adjustment for these phytoestrogens did not alter the associations between fiber and breast cancer risk (data not shown).

Some limitations need to be considered when interpreting our results. The FFQ was not validated in different racial/ethnic groups. Therefore, it is not known whether there are differences in validity between racial/ethnic groups and whether this explains why we observed different associations by race/ethnicity. The dietary assessment was based on self‐report, and inaccurate recall of past dietary intake might have introduced some exposure misclassification. Although we cannot rule out the possibility of differential recall of dietary intake by case–control status, it is unlikely that such recall bias would differ by ER/PR status among cases. The FFQ did not consider fiber intake from supplements. Fiber supplements, however, contribute little to total fiber intake 59. Lastly, we could only examine the association with recent consumption of fiber and fiber‐rich foods. There is some evidence that adolescent dietary fiber intake may reduce the risk of breast cancer 60, 61 and proliferative benign breast disease, a marker of increased breast cancer risk 62. A 34% reduction in breast cancer risk was associated with the highest quintile of adolescent fiber intake 61, suggesting that adolescent fiber intake may be an important exposure to consider.

This study has several strengths, including the large sample size and racially/ethnically diverse population. The large number of Hispanic participants allowed us to assess a wider range of fiber intake than if the study had included NHW women only. The FFQ assessed both frequency of consumption and usual portion size to estimate usual intake. To facilitate recall of portion size, food models and utensils were used. We were able to adjust the analyses for many known risk factors for breast cancer, which was found to be important as the inverse associations were somewhat attenuated after multivariate adjustment. Lastly, information on ER and PR status was available for most cases (90%). Prior studies that did not consider tumor hormone receptor status may have failed to detect inverse associations with intake of fiber or fiber‐rich foods.

The finding of inverse associations of ER‐PR‐ breast cancer with intake of bean fiber, beans, and grains, if confirmed, has important public health implications. Few risk factors have been identified for ER‐PR‐ breast cancer which is more common in African American and Hispanic women and has worse prognosis. The identification of modifiable lifestyle factors is therefore particularly important for the prevention of this aggressive breast cancer subtype. The role of diet, including fiber, in the etiology of specific breast cancer subtypes, warrants further investigation.

There is increasing evidence that higher levels of acculturation into mainstream American culture by Hispanic women are often accompanied by health behaviors that may increase their risk of cancer, including the adoption of U.S. diets higher in fat and lower in fruits and vegetables 63, 64, 65. Our data for control women show that the consumption of fiber, and bean fiber in particular, is lower among U.S.‐born than foreign‐born Hispanics. If bean fiber is indeed effective in lowering breast cancer risk, it may contribute to the higher breast cancer risk among U.S.‐born Hispanics compared to foreign‐born Hispanics 25, 66.

Given that for a large proportion of women in our study, daily total fiber intakes fell short of daily recommended values, educational programs aimed at increasing intake of fiber or fiber‐rich foods such as beans and grains may be effective in reducing the risk of not only ER‐PR‐ breast cancer, but also other cancers, such as colon, rectal, and esophageal cancer 1, 67.

## Conflict of Interest

The authors declare that they have no conflict of interest.
